# White Matter Network Disruption and Cognitive Dysfunction in Neuromyelitis Optica Spectrum Disorder

**DOI:** 10.3389/fneur.2018.01104

**Published:** 2018-12-17

**Authors:** Eun Bin Cho, Cheol E. Han, Sang Won Seo, Juhee Chin, Jeong-Hyeon Shin, Hye-Jin Cho, Jin Myoung Seok, Sung Tae Kim, Byoung Joon Kim, Duk L. Na, Kwang-Ho Lee, Joon-Kyung Seong, Ju-Hong Min

**Affiliations:** ^1^Department of Neurology, Gyeongsang Institute of Health Science, Gyeongsang National University Changwon Hospital, Gyeongsang National University School of Medicine, Jinju, South Korea; ^2^School of Biomedical Engineering, Korea University, Seoul, South Korea; ^3^Department of Electronics and Information Engineering, Korea University, Sejong, South Korea; ^4^Department of Neurology, Samsung Medical Center, Sungkyunkwan University School of Medicine, Seoul, South Korea; ^5^Department of Neurology, Neuroscience Center, Samsung Medical Center, Seoul, South Korea; ^6^Department of Neurology, Bucheon St. Mary's Hospital, College of Medicine, The Catholic University of Korea, Bucheon, South Korea; ^7^Department of Neurology, Soonchunhyang University College of Medicine, Cheonan Hospital, Cheonan, South Korea; ^8^Department of Radiology, Samsung Medical Center, Sungkyunkwan University School of Medicine, Seoul, South Korea

**Keywords:** neuromyelitis optica spectrum disorder, cognitive dysfunction, white matter network, diffusion tensor imaging, graph theory, multiple sclerosis

## Abstract

**Background:** In neuromyelitis optica spectrum disorder (NMOSD), brain involvement is common and cognitive dysfunction is frequently found. The study investigated alterations of white matter (WM) connectivity using graph theory and correlations with cognitive dysfunction in patients with NMOSD.

**Methods:** We prospectively enrolled patients with NMOSD (*N* = 14) and age- and sex-matched healthy controls (*N* = 21). Structural connections between any pair of the 90 cortical and subcortical regions were established using diffusion tensor imaging and graph theory. Network-based statistics was employed to assess differences in WM connectivity between the NMOSD and healthy control groups. We further investigated the relationship between the topological network characteristics and cognitive test performances.

**Results:** WM network analysis showed decreased total strength of brain networks and two disrupted sub-networks in patients with NMOSD. The first featured six hub nodes in the rectus, hippocampus, calcarine, cuneus, and precuneus with the left-sided predominance. The second had six hub nodes in the orbitomiddle frontal, post-central, superior parietal, superior, and middle temporal, and caudate with the right-sided predominance. Compared to healthy controls, NMOSD patients showed poor performance on tests for attention/working memory and processing speed, visuospatial processing, and executive function, which were associated with significant decreases in nodal clustering coefficient, local efficiency, and regional efficiency in the disrupted sub-networks (all *p* < 0.05).

**Conclusions:** The data show the overall WM disruption and the relationship between poor cognitive function and sub-network alterations identified by the network analysis in NMOSD patients. We suggest that cognitive dysfunction is related to dysconnectivity of WM network including default mode network in NMOSD.

## Introduction

Neuromyelitis optica spectrum disorder (NMOSD) is an inflammatory demyelinating disease of central nervous system (CNS), which is characterized by the presence of anti-aquaporin4 antibody (AQP4-Ab) and the predominant involvement of optic neuritis and myelitis. However, brain involvement is frequently observed, particularly in 52–89% of patients with AQP4-Ab (1). Moreover, brain abnormalities in NMOSD patients were identified even in the normal-appearing white matter (WM) and normal-appearing gray matter (GM), through diffusion tensor imaging (DTI), magnetization transfer, and volumetric MR imaging (2–4). Previous studies have shown multiregional WM disruption in NMOSD, using DTI parameters (5, 6). In addition, recent studies reported that cognitive deficits were observed in 57–67% of patients with NMOSD with a similar cognitive profile to that of patients with multiple sclerosis (MS), which features poor performance on tests of complex attention, executive function, speed of information processing, and/or memory (7–10). Brain volumetric MR studies of NMOSD reported the correlation between impaired cognition and WM damage focusing on WM atrophy (7, 11, 12). Still, unanswered question remains as to WM connectivity changes affect the cognitive functions in patients with NMOSD.

The human brain can be modeled as a complex structural network based on graph theory. According to this theory, brain networks can be described as graphs composed of nodes (neurons or brain regions) linked by edges (synapses or white matter connections), and large-scale white matter connectivity can be constructed using DTI (13). The healthy brain exhibits an efficient “small-world” property, which combines high levels of local clustering among nodes of the network (local segregation or specialization) and short average path lengths that represent global linkage of all nodes in the network (global integration) (13). There is growing evidence that normally efficient brain networks are affected in various diseases in a disease-specific manner (14, 15). To our knowledge, only one study has examined the altered topological organization of white matter connectivity in patients with NMO using graph theoretical analyses. Structural networks were constructed in each NMO patient and control group and regional efficiencies of the common hub nodes were compared (3). However, the clinical implication of WM network changes related to cognitive dysfunction remains unknown.

In this study, we investigated the topological organization of whole brain WM networks by comparing patients with NMOSD and healthy controls. Given multiregional WM damages were reported in previous studies in NMOSD, we adopted three hypotheses. The first is that there are topological alterations of WM networks in patients with NMOSD. The second is that there is a set of sub-networks that distinguish patients from healthy controls. The third is that topological characteristics of these sub-networks are related to impaired cognition.

## Materials and Methods

### Subjects

A total of 15 patients with NMOSD and 22 healthy controls were enrolled in this prospective, single tertiary center study between January 2012 and December 2013. One patient from either group was excluded because of inadequate build-up of tractography due to head motion and blurring of MRI. Finally, 14 patients with NMOSD (13 females, one male; median age, 39 years; range 21– 68) and 21 healthy controls (20 females, one male; median age, 30 years; range 22–56) were included. AQP4-Ab was measured with a cell-based indirect immunofluorescence assay as previously described (16). Thirteen patients (93%) had anti-AQP4-Ab and six patients (43%) met the diagnostic criteria for NMO (17). At the time of inclusion, all patients were in remission for more than 3 months and nine patients (64%) had received oral prednisolone at a median dose 10.0 mg/day (range, 2.5–10). Healthy controls did not have any history of medical, neurological, or psychiatric disorders. The present study was approved by the Institutional Review Board of Samsung Medical Center and written informed consent was obtained from all participants in accordance with the Declaration of Helsinki.

### Neuropsychological Tests

All participants underwent a comprehensive neuropsychological test battery assessing all cognitive domains suggested by the Rao Brief Repeatable Neuropsychological Battery (BRBN) (18) or the Minimal Assessment of Cognitive Function in Multiple Sclerosis (MACFIMS) (19). For attention/working memory and processing speed, we used the Digit Span Test forward and backward, Trail Making Test part A, Paced Auditory Serial Addition Test (PASAT) at 3 and 2 s, and Digit Symbol Coding Test (DSCT). For visuospatial/perceptual processing, the Spatial Span forward and backward tests were used. We used the Korean version of Boston Naming Test (BNT) (20) to evaluate confrontational naming ability as language function and the Rey-Osterrieth Complex Figure Test (RCFT) to evaluate visuospatial function. In addition, the Korean version of California Verbal Learning Test (CVLT) (20) and the immediate recall, delayed recall, and recognition trials of RCFT were performed to evaluate memory (animal and supermarket items), the Korean version of Controlled Oral Word Association Test (COWAT) using the three Korean letters, which were compatible with the English version of “F,” “A,” and “S” (21), and Trail Making Test (TMT) part B. Moreover, considering mood effects on neuropsychological testing, we evaluated the depressive symptomatology and anxiety with the Beck Depression Inventory-II (BDI-II) (22) and State-Trait Anxiety Inventory (STAI) (23), respectively. The time interval between neuropsychological tests and brain MRI was within a month.

### Image Acquisition

All participants underwent a three-dimensional (3D) volumetric brain MRI scan. An Achieva 3.0-Tesla MRI scanner (Philips, Best, the Netherlands) was used to acquire 3D T1 Turbo Field Echo (TFE) MRI data using a sagittal slice thickness of 1.0 mm, over contiguous slices with 50% overlap and no gap, a repetition time (TR) of 9.9 ms, an echo time (TE) of 4.6 ms, a flip angle of 8° and matrix size of 240 × 240 pixels reconstructed to 480 × 480 over a field of view of 240 mm. 3D FLAIR MRI data were acquired in the axial plane with the following parameters: axial slice thickness of 1 mm, no gap; TR of 11000.0 ms; TE of 125.0 ms; flip angle of 90°; and matrix size of 512 × 512 pixels. In the whole-brain diffusion weighted MR imaging (DWI), sets of axial diffusion-weighted single-shot echo-planar images were collected with the following parameters: 128 × 128 acquisition matrix; 1.72 × 1.72 × mm^3^ voxels; 70 axial slices; 220 × 220 mm^2^ field of view; TE 60 ms, TR 7696 ms; flip angle 90°; slice gap 0 mm; b-factor of 600 s/mm^2^. With the baseline image without diffusion weighting (the reference volume), DWI were acquired from 45 different directions. All axial sections were acquired parallel to the anterior commissure−posterior commissure line and perpendicular to the mid-sagittal plane.

### Image Preprocessing and Network Construction

We used the automated anatomical labeling (AAL) template (24), which contains 78 cortical regions and 12 subcortical structures. For each subject, we non-linearly registered her/his T1 weighted image to the ICBM152 T1 template in the MNI space where the AAL template is defined, and linearly registered the T1 weighted image to her/his own DWI (FSL, http://www.fmrib.ox.ac.uk/fsl/). We then mapped the anatomically defined regions-of-interest (ROIs) defined in the AAL template to the individuals' diffusion space. We used the nearest neighbor interpolation method to preserve the discrete labels of the AAL temple during this inverse mapping.

To obtain streamline tractography, we first performed eddy-current correction on DWI by registering all volumes with non-collinear diffusion directions to the reference image using FSL. Then we employed the Fiber Assignment by Continuous Tracking (FACT) algorithm (25) with 45 degrees of angle threshold through the Diffusion toolkit along with TrackVis (26). This program performed tractography from all voxels (seed voxels) of WM whose fractional anisotropy (FA) value is over 0.2, except ventricles. We removed streamlines shorter than 20 mm in length.

Connectivity matrices were obtained by averaging FA values following neural tracts connecting between any two regions of interests (ROIs) (27). The FA values represent the WM integrity where the lower values may be associated with the damage on the WM in the patient group (28). Specifically, we collected all voxels passed by all streamlines which connect two ROIs, and averaged their FA values. The resulting matrix contains the mean FA values between all pairs of ROIs as its weight. This FA-weighted connectivity matrix may incorporate the WM damage in NMOSD better than mere streamline counts.

### Network Topology Analysis

Network topological measures were computed using the Matlab routines of the Brain Connectivity Toolbox (29). Global topological measures included total strength (the summation of all weights in each subject's brain network), edge density (the number of non-zero edges over the number of all possible edges), clustering coefficients, characteristic path length, and small-worldness (29). The values of the last three measures are related to the edge density (30). Thus, we normalized them with the 100 randomly generated degree-strength preserved random networks (31). The clustering coefficient captures the level of local segregation. For each node, nodal clustering coefficient measures how strongly its neighbored nodes are connected each other. The global measure of clustering coefficient is the average clustering coefficient across all the nodes. The characteristic path length is the harmonic mean of all shortest paths capturing the global integration. The small-worldness captures the balance between global integration and local segregation. It is meaningful only when it is compared to the random networks; the characteristic path length is similar to that of the random network, while the clustering coefficient is far larger than that of the random network. Thus, it is given by the value of the normalized clustering coefficient over the normalized characteristic path length; when it is bigger than 1, the network is considered as a smallworld network.

We used nodal measures including nodal degree, nodal strength, nodal clustering coefficient, local efficiency, participation coefficients, and regional efficiency. All of them measure the centrality of a node in various aspects. The nodal degree and strength captures how many neighbors a node is connected with and how strongly it is connected with its neighbors in order. Specifically, the former is the number of edges that connected to the node, while the latter is the summation of the edges' strengths in terms of the number of streamlines. The local efficiency is similar concept with the clustering coefficient, which captures the efficiency of information communication between the neighbors of a certain node (32). The participation coefficient of a node captures its role in the modular organization (33). The brain network in general have a modular structure which consists of several modules whose intra-modular connectivity is denser than their inter-modular connectivity. The higher value represents that the node is connected with multiple modules and may have an important role in exchanging information between modules. The regional efficiency also captures the level of information exchanges in different levels. The regional efficiency summarizes how efficiently information of a node can be exchanged with all the other nodes by averaging reciprocals of the shortest path lengths to all the other nodes (34).

## Statistical Analyses

Mann-Whitney *U* test and Chi-square test were performed to compare demographic variables and neuropsychological test scores between the NMOSD and healthy control groups. The Bonfferoni correction was performed over all 22 neuropsychological test scores for the multiple comparison correction.

For comparison of the global network measures, we used permutation-based ANCOVA, controlling for age, sex, and the duration of education. We re-populated the data sets *N-1* times by random re-assignment (permutation) of all subjects into one of two groups, where *N* is the number of permutations. We computed *F*-values for the original data set and *N-1* permuted sets through a simple ANCOVA, which formed a null distribution of group difference. Then we estimated the significance level of group difference by a fraction of the occurrence whose *F*-values were not less than the *F*-value of the original data set. We used 10,000 as *N*. We then employed the Bonferroni correction for 5 network measures as multiple comparison correction. For the survived network measures, we performed the correlation study with each score of neuropsychological test items of impaired performance using the partial correlation coefficients controlling for age, sex, and the duration of education.

For comparison of WM connectivity, we first performed two-sample *t*-test for each edge between the controls and the NMOSD patients, and then employed the network-based statistics (NBS) analysis for multiple comparison correction (35). NBS extracted sub-networks that consisted of significantly different connections between two groups. Specifically, significance levels of sub-networks were estimated based on how the size of the sub-networks was bigger than randomly formed sub-networks using permutation testing. In other words, a sub-network was defined as a set of connected edges whose representative statistics (i.e., *t*-statistics) was bigger than a certain threshold. A value of 2.5 was used as the initial threshold and 10,000 as the number of permutations. NBS works as multiple comparison correction, by controlling the family-wise error rate in the weak sense.

The relationships between the network topological characteristics of “hub nodes” in the sub-network and the clinical variables in patients with NMOSD were examined using Spearman correlation analysis. The hub nodes represented the brain regions most affected by the identified abnormal structural connectivity, and were defined when their degree exceeded the mean plus two standard deviations over all regions connected by the edges identified by NBS. The nodal measures for the correlation study with the neuropsychological tests include nodal strength, nodal clustering coefficient, participation coefficients, local efficiency, and regional efficiency. The clinical variables used in this analysis were part of cognitive function tests that exhibited differences between two groups with Bonferroni adjusted *p* < 0.05. Since the network measures often do not follow the normal distribution, Spearman partial correlation was used to control for the effects of age, gender, and level of education. The false discovery rate (FDR) procedure was performed for the multiple comparison correction over all found hub nodes (i.e., 12 nodes). While the correlation study of the global network measures showed how the global network organization affects the cognitive performance, the correlation study of the nodal network measures in the abnormal WM network may capture how the local topological changes due to the disease affect the cognitive function.

The demographic values and neuropsychological tests were analyzed through SPSS Version 20.0 (IBM Corp, Armonk, NY, USA). All other statistical analyses and visualization were performed using Matlab R2013a (Mathworks, Natick, MA, USA) and in-house software programs.

## Results

### Demographic and Clinical Features

Demographic and clinical features of 14 NMOSD patients and 21 healthy controls are summarized in Table 1. Sex ratio, age, and duration of education did not differ between patients with NMOSD and healthy controls. In NMOSD patients, the most frequent involvement was the spinal cord (*N* = 9, 62.4%), followed by the optic nerve (*N* = 6, 42.9%), brainstem/cerebellum (*N* = 6, 42.9%), and cerebral hemisphere (*N* = 2, 14.3%). No patient showed gadolinium-enhancement in cerebral hemispheric lesions.

**Table 1:** Demographics and clinical characteristics of study subjects.

	**NMOSD**	**HC**	***P*-value**
	**(*n* = 14)**	**(*n* = 21)**	
Sex, female (%)	13 (92.9)	21 (95.5)	1.000
Age, years (range)	39 (21–68)	30 (22–56)	0.092
Education (%)			0.200
<9 years	2 (14.3)	0 (0)	
9–14 years	5 (35.7)	8 (38.1)	
≥15 years	7 (50.0)	13 (61.9)	
Positive AQP4 Ab (%)	13 (93)	NA	
Definite NMO (%)	6 (43)	NA	
Disease duration, years	3.4 (1.5–9.3)	NA	
Annual relapse rate	0.6 (0.5-1.7)	NA	
Anytime involvement (%)		NA	
Cerebral hemisphere^a^	2 (14.3)	NA	
Brainstem/cerebellum^a^	6 (42.9)	NA	
Spinal cord	9 (64.3)	NA	
Optic nerve	6 (42.9)	NA	
EDSS	3.0 (1.375–5.25)	NA	

*NMOSD, neuromyelitis optica spectrum disorder; HC, healthy control; EDSS, Expanded Disability Status Scale*.

*Values are median (IQR, interquartile range) unless otherwise indicated; NA, Non Assessable*.

a *Symptomatic or asymptomatic brain lesions on MRI for this study; frontoparietal white matter lesions in 2/14 (14.3%) and area postrema/cerebellum lesions in 6/14 (42.9%)*.

### Cognitive Dysfunction in NMOSD Patients

Compared to healthy controls, patients with NMOSD showed significant differences for the following tests scores: attention/working memory and processing speed (TMT-A, corrected *p* = 0.001; PASAT3, corrected *p* = 0.004; PASAT2, corrected *p* = 0.007; and DSCT, corrected *p* = 0.001), executive function (TMT-B, corrected *p* = 0.022; COWAT, corrected *p* = 0.006), and visuospatial processing (spatial span backward, corrected *p* = 0.022) (Table 2; Table e-1). Digit span forward and backward test in attention/working memory, RCFT copy in visuospatial function, and K-CVLT and RCFT in verbal and visual memory were not different between the NMOSD and healthy control groups.

**Table 2:** Neuropsychological test results from patients with NMOSD and healthy control group.

**Neuropsychological test**	**NMOSD**	**Healthy control**	***P*-value^**a**^**
**(possible maximum score)**	**(*n* = 14)**	**(*n* = 21)**
**ATTENTION/WORKING MEMORY AND PROCESSING SPEED**			
Digit span forward (12)	8.5 (6.75–10.25)	11 (8–12)	0.770
Digit span backward (12)	6 (5–8)	8 (6–11)	0.946
Trail Making Test A (sec)	31 (29.5–58.5)	21 (16–24.5)	**0.001**
PASAT 3” (60)	39.5 (26.75–49.75)	56 (50–59)	**0.004**
PASAT 2” (60)	26.5 (17.5–37.5)	43 (39–50.5)	**0.007**
Digit symbol coding test (90)	58 (31.5–71.5)	80 (78–90)	**0.001**
**VISUOSPATIAL/PERCEPTUAL PROCESSING**			
Spatial span forward (14)	8 (7–10)	11 (9.5–12)	0.110
Spatial span backward (12)	8 (6–8.5)	9 (8.5–10)	**0.022**
**LANGUAGE**			
K-BNT (60)	51 (46–54)	54 (51–56)	0.418
**VISUOSPATIAL FUNCTION**			
RCFT copy (36)	35 (33–36)	36 (34–36)	1.000
**VERBAL MEMORY**			
K-CVLT Immediate recall (36)	54 (44–59.5)	59 (52–62)	1.000
K-CVLT Delayed recall (16)	12 (10.75–14)	14 (11–14.5)	1.000
K-CVLT Recognition (16)	15 (12.75–16)	15 (15–16)	1.000
**VISUAL MEMORY**			
RCFT Immediate recall (36)	16.5 (7–22.75)	21 (18–27.25)	0.528
RCFT Delayed recall (36)	14 (9.75–23.5)	20.5 (16.25–24.75)	1.000
RCFT Recognition (24)	20 (18–21)	20 (19–22)	1.000
**EXECUTIVE FUNCTION**			
Trail Making Test B (sec)	83 (63.25–138.75)	53 (42.5–59)	**0.022**
Semantic generative naming	36 (25.75–38.75)	41 (36.5–45)	0.154
COWAT phonemic	26.5 (15.5–42)	52 (39–57.5)	**0.006**
**EMOTION**			
BDI-II (63)	11.5 (7.75–24.25)	5 (3–7)	0.066
STAI_state (80)	44.5 (34–59.5)	36 (32–44)	1.000
STAI_trait (80)	44.5 (34–63.25)	37 (29–44)	1.000

*NMOSD, neuromyelitis optica spectrum disorders; PASAT, Paced Auditory Serial Addition Test; K-BNT, Korean version of the Boston Naming Test; K-CVLT, Korean-California Verbal Learning Test; RCFT, Rey-Osterrieth Complex Figure Test; COWAT, Controlled Oral Word Association Test; BDI-II, Beck Depression Inventory-II; STAI, State-Trait Anxiety Inventory*.

*Data area expressed as median (interquartile range)*.

a *P-values are results of Mann-Whitney U test adjusting through Bonferroni correction over 22 different test scores (bold indicates the significant results)*.

The frequency of moderate depression or more as BDI ≥ 19 was higher in patients with NMOSD (*N* = 5, 35.7%), compared to healthy controls (*N* = 1, 5%), although BDI-II score was not statistically different between patients with NMOSD and healthy controls.

### Correlations Between Cognitive Test Performance and Clinical Parameters in NMOSD Patients

No significant correlation was observed between cognitive test performances and clinical parameters, such as disease duration, annualized relapse rate, and EDSS scores, in NMOSD patients. The location of symptomatic lesions (brain, spinal cord, or optic nerve) was not associated with cognitive test performance. The dosage of oral prednisolone was not associated with depressive mood and cognitive performances in NMOSD patents. However, depressive mood was positively correlated with PASAT 2 and TMT-B scores (ρ = −0.696; *p* = 0.037 and ρ = 0.729; *p* = 0.021, respectively) in NMOSD patients, after controlling for age, sex, and education level.

### Global Network Topology Analysis

Compared to healthy controls, the total strength was lower in NMOSD patients (*p* = 0.009), although no significant differences were found in edge density, normalized clustering coefficient, and normalized characteristic path length between the two groups (Table 3). Both brain networks of NMOSD patients and healthy controls showed the small-world characteristics (small-worldness > 1), which was not different between the two groups.

**Table 3:** Global network topological measures.

**Network topological measures**	**NMOSD**	**HC**	***P*-value^**a**^**
Total strength	275.03 ± 31.61	301.40 ± 22.50	**0.045**
Edge density	0.173 ± 0.011	0.175 ± 0.010	1.000
Clustering coefficient^**b**^	2.164 ± 0.190	2.132 ± 0.170	1.000
Characteristic path length^**b**^	1.073 ± 0.027	1.072 ± 0.016	1.000
Small-worldness^b^	2.015 ± 0.145	1.987 ± 0.145	1.000

*NMOSD, neuromyelitis optica spectrum disorders; HC, healthy controls*.

*Data area expressed as mean ± standard deviation*.

a *P-values are results of the permutation test adjusting through Bonferroni correction over all 5 network measures (bold indicates the significant results)*.

b *Normalized value*.

### Disrupted WM Connectivity in NMOSD Patients

**Figure 1:**
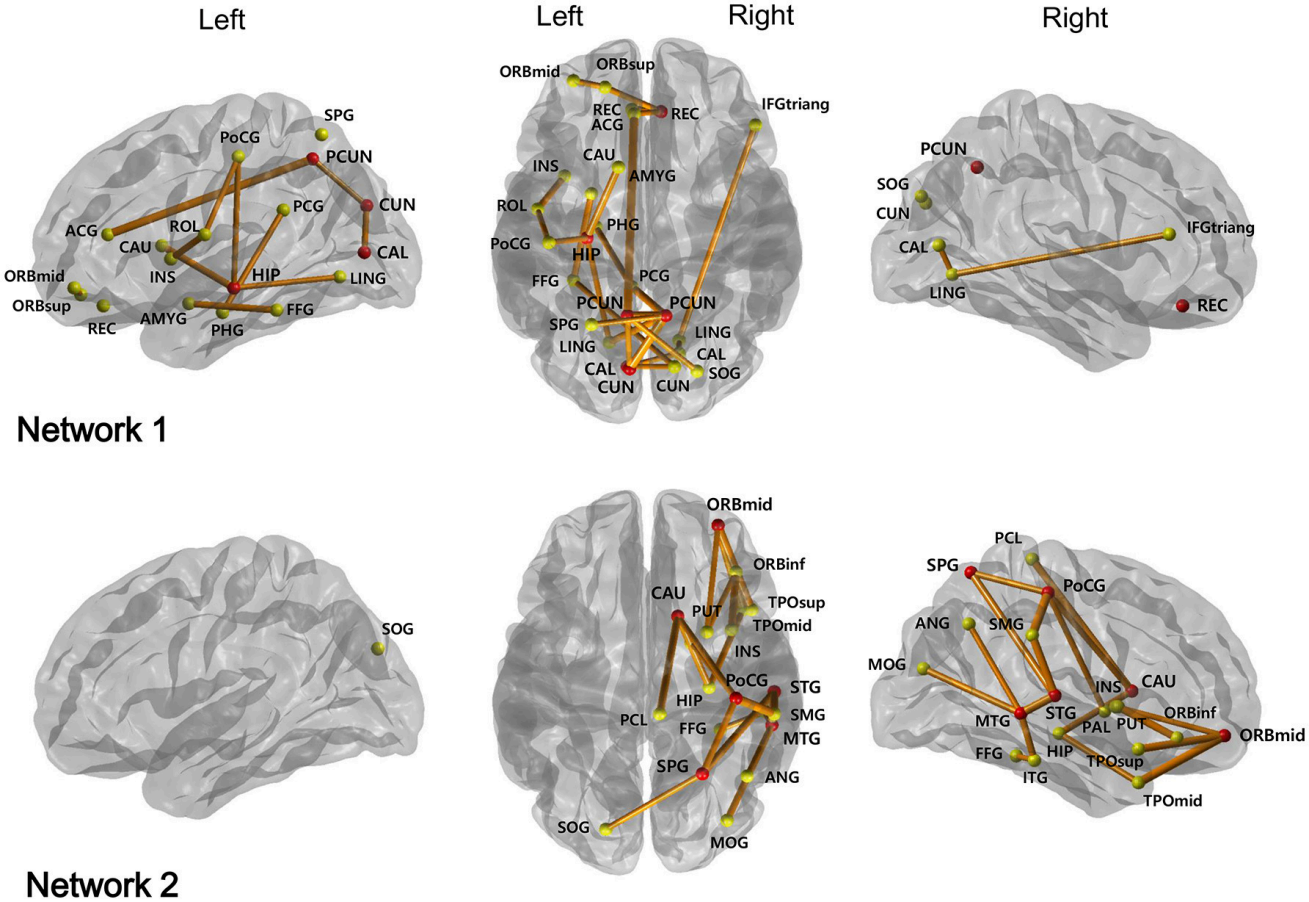
Two subnetworks were identified by network-based statistics, which show the reduced connectivities in NMOSD patients compared to healthy controls. The red circles represent their hub regions representing the brain regions most affected by the white matter disruption, while the other yellow circles are the non-hub brain regions. ORBsup, Superior frontal gyrus, orbital part; ORBmid, Middle frontal gyrus, orbital part; REC, Rectus; ROL, Rolandic operculum; INS, Insula; ACG, Anterior cingulum; PCG, Posterior cingulum; PHG, Parahippocampal gyrus; CAL, Calcarine; CUN, Cuneus; HIP, Hippocampus; LING, Lingual gyrus; IFGtriang, Inferior frontal gyrus, triangular part; AMYG, Amygdala; FFG, Fusiform gyrus; PoCG, Post-central gyrus; SOG, Superior occipital gyrus; PCUN, Precuneus; SPG, Superior parietal gyrus; CAU, Caudate; ORBinf, Inferior frontal gyrus, orbital part; SMG, Supramarginal gyrus; PCL, Paracentral lobule; PUT, Putamen; PAL, Pallidum; STG, Superior temporal gyrus; TPOsup, Temporal pole: middle temporal gyrus; MTG, Middle temporal gyrus; MOG, Middls occipital gyrus; ANG, Angular gyrus; TPOmid, Temporal pole: middle temporal gyrus; ITG, Inferior temporal gyrus.

NBS analysis identified two disrupted sub-networks in NMOSD patients compared to healthy subjects (Table 4, Figure 1). All found connections in the sub-networks have weaker strengths in the NMOSD patients. The first sub-network consisted of regions located in the limbic and parieto-occipital lobes, predominantly in the left and posterior part of the brain. These regions were heavily connected to six hub nodes including the right rectus, left hippocampus, left calcarine, left cuneus, and bilateral precuneus. The hub nodes were heavily connected with the disrupted connections more than the average; in this manner, they represented the brain regions most affected by the identified abnormal structural connectivity. The second sub-network included regions mostly within the right temporal, parietal, orbitofrontal lobes, and basal ganglia. These regions were heavily connected to six hub nodes: the right orbitomiddle frontal gyrus, right post-central gyrus, right superior parietal gyrus, right caudate, and right superior and middle temporal gyrus.

**Table 4:** The subnetworks of reduced connectivity in NMOSD patients identified through network-based statistics (NBS).

**Subnetwork 1**	**T-stat ^**a**^**	**Subnetwork 2**	**T-stat^**a**^**
Lt. superior frontal, orbital part—Lt. middle frontal, orbital part	2.820	Rt. inferior frontal, orbital part—Rt. insula	3.450
Lt. superior frontal, orbital part—**Rt. rectus**^**b**^	2.798	Rt. inferior frontal, orbital part—Rt. putamen	2.507
Lt. rectus—**Rt. rectus**^**b**^	3.399	**Rt. middle frontal, orbital part**^**b**^—Rt. putamen	2.617
Lt. rolandic operculum—Lt. insula	2.996	**Rt. middle frontal, orbital part**^**b**^—Rt. temporal pole, superior temporal	3.550
**Rt. rectus**^**b**^—Lt. anterior cingulum	2.525	**Rt. middle frontal, orbital part**^**b**^—Rt. temporal pole, middle temporal	4.011
Lt. posterior cingulum—Lt. parahippocampus	2.794	**Rt. post-central**^**b**^—**Rt. superior parietal**^**b**^	2.949
**Lt. hippocampus**^**b**^—Lt. lingual	2.606	**Rt. post-central**^**b**^—Rt. supramarginal	4.064
**Lt. hippocampus**^**b**^—Lt. postcentral	2.662	**Rt. post-central**^**b**^—**Rt. caudate**^**b**^	2.770
**Lt. hippocampus**^**b**^—Lt. caudate	3.272	**Rt. post-central**^**b**^—Rt. pallidum	3.358
Rt. inferior frontal, triangular part—Rt. Lingual	2.839	Lt. superior occipital—**Rt. superior parietal**^**b**^	2.714
Rt. calcarine—Rt. Lingual	2.502	**Rt. superior parietal**^**b**^—**Rt. superior temporal**^**b**^	3.358
Lt. amygdala—Lt. fusiform	3.088	Rt. hippocampus—**Rt. caudate**^**b**^	3.040
**Lt. calcarine**^**b**^—Rt. Calcarine	2.521	Rt. paracentral lobule—**Rt. caudate**^**b**^	3.449
**Lt. calcarine**^**b**^—**Lt. cuneus**^**b**^	2.834	Rt. supramarginal—**Rt. superior temporal**^**b**^	3.568
**Lt. calcarine**^**b**^—Rt. Cuneus	2.587	**Rt. superior temporal**^**b**^—**Rt. middle temporal**^**b**^	3.843
Rt. cuneus—Lt. fusiform	2.564	Rt. middle occipital—**Rt. middle temporal**^**b**^	3.145
Lt. rolandic operculum—Lt. post-central	2.519	Rt. angular—**Rt. middle temporal**^**b**^	2.699
Lt. anterior cingulum—**Lt. precuneus**^**b**^	3.754	**Rt. middle temporal**^**b**^—Rt. inferior temporal	3.112
**Lt. cuneus**^**b**^—**Lt. precuneus**^**b**^	2.615	Rt. hippocampus—Rt. temporal pole, middle temporal	2.944
Rt. superior occipital—**Lt. precuneus**^**b**^	2.717	Rt. fusiform—Rt. inferior temporal	2.576
Lt. posterior cingulum—**Rt. precuneus**^**b**^	3.456		
**Lt. cuneus**^**b**^—**Rt. precuneus**^**b**^	3.838		
Lt. lingual—**Rt. precuneus**^**b**^	3.175		
Lt. superior parietal gyrus—**Rt. precuneus**^**b**^	2.641		
**Lt. precuneus**^**b**^—**Rt. precuneus**^**b**^	3.830		

a *T-statistics (T-stat) is a result of two-sample t-test. NBS was used as a multiple comparison correction, with 2.5 as a threshold value and 10,000 permutations*.

b *Hubs of the subnetworks 1 and 2 were marked as bold*.

*NMOSD, neuromyelitis optica spectrum disorders; Lt., left; Rt., right*.

### Cognitive Test Performance Associated With the Disrupted WM Connectivity in NMOSD Patients

We found that the total strength was positively associated with PASAT3 scores (*r* = 0.712, uncorrected *p* = 0.014) in NMOSD patients. Significant correlation was evident between topological characteristics of the hub nodes and cognitive test performances, which differed significantly between the two groups (Table 5). The nodal clustering coefficient of the right superior temporal gyrus was positively correlated with TMT-B test performance (*r* = −0.893, FDR adjusted *p* = 0.014). The reduced local efficiency of the left calcarine, cuneus, and right precuneus and the reduced regional efficiency of the left calcarine were associated with decreased performance on DSCT (all FDR adjusted *p* < 0.05). In addition, the participation coefficient of the right post-central gyrus was positively associated with PASAT3 scores (*r* = 0.795, FDR adjusted *p* = 0.041).

**Table 5:** The topological characteristics of hub nodes in association with cognitive test performance in patients with NMOSD.

**Topological characteristics**	**Hub nodes**	**Neuropsychological tests**	***R* values (adjusted *p*-values)^**a**^**
Nodal clustering coefficient	Rt. Temporal_Sup	TMT-B	−0.8934 (0.0140)
Local efficiency	Lt. Calcarine	DSCT	0.9122 (0.0028)
	Lt. Cuneus	DSCT	0.8405 (0.0139)
	Rt. Precuneus	DSCT	0.7817 (0.0303)
Regional efficiency	Lt. Calcarine	DSCT	0.8430 (0.0263)
Participation coefficient	Rt. Post-central	PASAT3	0.7951 (0.0413)

*NMOSD, neuromyelitis optica spectrum disorders; Temporal_Sup, superior temporal gyrus; TMT-B, Trail Making Test-B; DSCT, Digit Symbol Coding Test; PASAT3, Paced Auditory Serial Addition Test at 3 s*.

a *Partial correlation coefficient analysis adjusting age, sex, and duration of education with FDR correction for the multiple comparison correction over all 12 hub nodes*.

## Discussion

We demonstrate the dysconnectivity of overall WM network and two disrupted sub-networks in patients with NMOSD. Moreover, the strength of global WM network and several topological characteristics of hub nodes in sub-networks were closely related to cognitive dysfunction, especially for attention/working memory, processing speed and executive function.

In the global WM network, the total strength of patients with NMOSD was decreased, but other topological measures were not different, compared to healthy controls. This is not consistent to a previous study, where the structural brain networks of NMO patients exhibited a disrupted topological organization (abnormal small-world parameters) without changes in network strength and efficiencies compared to healthy controls (3). They previously investigated the WM networks of MS using similar analytical method and found reduced network strength, global and local efficiencies (15); therefore, they suggested that greater damage to brain tissue in MS than in NMO could explain the different changes in WM structural brain networks (3, 15). Other studies using DTI and tract-based spatial statistics in NMO patients showed extensive cerebral WM tracts with decreased FA and increased mean diffusivity (MD) value, although patients had no brain lesions or small and non-specific lesions (5, 6). These suggested the possibility of widespread non-lesional pathology in normal appearing WM of NMO patients. In our study, although most of patients had normal brain MRI without severe tissue damage, the total strength was decreased, which may imply that the overall WM dysconnectivity results from the disruption of the two sub-networks by non-lesional change, without the alteration of architecture in global network.

More interestingly, we found 12 hub nodes in two disrupted sub-networks in NMOSD patients, of which 6 were regions in the default mode network (DMN); the bilateral precuneus, right rectus, and left hippocampus, right middle and superior temporal gyri (36). The concept of DMN comes from resting-state functional MRI, altered in MS and Alzheimer's disease (15, 36). Previously, the functional alteration of DMN regions, such as the left anterior/posterior cingulate, left medial frontal gyrus, right precuneus and right middle temporal gyrus was observed in NMO patients (37, 38). Other 6 hub nodes in our study were non-DMN regions; left calcarine, left cuneus, right post-central gyrus, right superior parietal gyrus, right orbitomiddle frontal gyrus, and right caudate. A recent study also showed the decreased regional efficiencies of non-DMN regions such as the right cuneus and left calcarine in NMO patients (3).

Decreased cognitive performance in our patients, compared to controls, was observed in attention/working memory and processing speed, visuospatial processing, and executive function and frequent depressive mood, which echo previous studies (9, 10, 39). The negative association between depression and attention/working memory, processing speed and executive function, was also consistent to the other studies (8, 9). Regarding the relationship between the cognitive function and topological characteristics of WM network, several interesting features in our study are notable. First, the total strength of global WM network was associated with PASAT3 score, which indicates that the dysconnectivity of global WM network was related to decreased attention/working memory and processing speed. Second, topological characteristics of 5 hub nodes in the sub-networks were also associated with DSCT/PASAT3 scores or TMT-B score. Decreased local or regional efficiencies of right precuneus, the left calcarine and left cuneus were associated with poor performance on the DSCT, which suggests that the decreased local functional specialization or regional contribution to global integration in these regions were associated with decreased attention/working memory and processing speed. The precuneus, one of DMN regions, is known to be implicated in visuo-spatial imagery, episodic memory retrieval and self-processing operations, (40) and the cuneus and calcarine also include primary visual pathway contributing to visuospatial function (41). DSCT is highly correlated with symbol digit modality test (SDMT) (42), which is known as a very sensitive task of cognitive processing speed in MS and is also known that visual scanning efficiency explains ~34% of digit symbol variance independent of perceptual motor speed (43). Therefore, decreased performance on DSCT in our patients might be attributed to decreased connection integrity of these regions regarding visuospatial function, as well as decreased processing speed. In addition, decreased clustering coefficient in the right superior temporal gyrus was associated with poor performance on TMT-B, which implies that the local functional specialization of this region was associated with executive function. That seems to be plausible since the right superior temporal gyrus, another DMN region, plays a crucial role in spatial awareness, visual search and probably maintaining working memory (44, 45). Finally, participation coefficient in the right post-central gyrus had positive correlation with PASAT3 scores, suggesting that the strength of connections to other modules in this region was associated with attention, working memory and processing speed. This may support the previous study, where post-central gyrus was one of lesions affecting processing speed, measured in the digit symbol subset (46).

The association between cognitive impairment and dysfunction of the DMN, such as precuneus and superior temporal gyrus, as well as non-DMN regions like the right sensory cortex and superior parietal gyrus, was reported in MS patients (47, 48). However, there have been no studies for the relatinoship between network connectivity and cognitive function in NMO, although it was reported that cognitive impairment could be attributed to damage in WM tracts (8, 11), and the reduced regional WM volume and brain atrophy were correlated with cognitive impairment (8). To our knowledge, only one study has examined the altered topological organization of WM connectivity in patients with NMO using graph theoretical analyses (3). Our study has several strengths compared to the previous study. First, we associated the changes in the topological measures with the clinical presentation. Thus, instead of mere difference in the topological measures, we investigated associations between topological network changes and specific cognitive functions with comprehensive neuropsychological testing. Second, our study presented statistically more valid observations. Although, the previous study reported the local changes in the nodal efficiency whose uncorrected *p*-values were < 0.5, we employed the FDR procedures over the hubs nodes of the disrupted WM sub-networks for conceptualizing the rate of type I errors when conducting multiple comparisons.

We have several limitations. This is a cross-sectional study performed in a single center. We enrolled NMOSD patients who could have had a relatively mild disability to perform the cognitive tests, which may limit the generalization of this study to all NMOSD patients along with the small sample size. In addition, some of the confounders, such as fatigue and pain, which may influence the cognitive function, were not considered in this study. Moreover, DWI data is rather suboptimal with respect to fiber tracking; the voxel dimensions are anisotropic (2 mm vs. 1.72 mm), which can bias tracking accuracy in the longer z-direction. However, the bias might be insignificant, since it is only 16% longer than the other directions. Finally, our study inherited the limitation of DTI and the deterministic tractography, including the issue of crossing-fibers and false positive connections (49). Other tracking method such as probabilistic tractography or Hough transform global tractography could be employed in the future research.

In conclusion, this study identified the alteration of overall WM network and the integrity disruption of sub-networks, including both DMN and non-DMN regions, in patients with NMOSD. Two disrupted sub-networks were associated with the decreased attention/working memory, processing speed and executive function, which suggests that the dysconnectivity of WM network contributes to the cognitive dysfunction in NMOSD.

## Author Contributions

EBC, CH, J-KS, and J-HM contributed to the conception and design of the study. EBC, CH, SWS, JC, J-HS, H-JC, JMS, STK, BJK, DLN, KHL, J-KS, and J-HM contributed to the acquisition and analysis of data. EBC, CH, J-KS, and J-HM drafted the text and figures. All authors contributed to the review and editing.

### Conflict of Interest Statement

The authors declare that the research was conducted in the absence of any commercial or financial relationships that could be construed as a potential conflict of interest.
