# Supplementary material for: White Matter Network Disruption and Cognitive Dysfunction in Neuromyelitis Optica Spectrum Disorder
Source: Front Neurol. 2018 Dec 17;9:1104. doi: 10.3389/fneur.2018.01104 (PMC6304415; doi:10.3389/fneur.2018.01104)
Supplement: Supplementary file 1 [file Table_1.DOCX]

**Table e-1** Neurophysiological test results from NMOSD patients

| Neuropsychological test  (possible maximum score) | Test scores | Poor performance, N (%) | |
| --- | --- | --- | --- |
|  |  | < 1SD | < 2SD |
| **Attention/working memory**  **and processing speed** |  |  |  |
| Digit span forward (12) | 8.5 (6.75-10.25) | 2 (14%) | 0 (0) |
| Digit span backward (12) | 6 (5-8) | 3 (21%) | 0 (0) |
| Trail Making Test A (sec) | 31 (29.5-58.5) | 6 (43%) | 3 (21%) |
| PASAT 3” (60) | 39.5 (26.75-49.75) | 6 (43%) | 4 (29%) |
| PASAT 2” (60) | 26.5 (17.5-37.5) | 7 (50%) | 6 (43%) |
| Digit symbol coding test (90) | 58 (31.5-71.5) | 5 (36%) | 3 (21%) |
| **Visuospatial/perceptual processing** |  |  |  |
| Spatial span forward (14) | 8 (7-10) | 2 (14%) | 1 (7%) |
| Spatial span backward (12) | 8 (6-8.5) | 3 (21%) | 1 (7%) |
| **Language** |  |  |  |
| K-BNT (60) | 51 (46-54) | 1 (7%) | 0 (0) |
| **Visuospatial function** |  |  |  |
| RCFT copy (36) | 35 (33-36) | 3 (21%) | 1 (7%) |
| **Verbal memory** |  |  |  |
| K-CVLT Immediate recall (36) | 54 (44-59.5) | 1 (7%) | 0 (0) |
| K-CVLT Delayed recall (16) | 12 (10.75-14) | 5 (36%) | 1 (7%) |
| K-CVLT Recognition (16) | 15 (12.75-16) | 8 (57%) | 1 (7%) |
| **Visual memory** |  |  |  |
| RCFT Immediate recall (36) | 16.5 (7-22.75) | 5 (36%) | 3 (21%) |
| RCFT Delayed recall (36) | 14 (9.75-23.5) | 6 (43%) | 2 (14%) |
| RCFT Recognition (24) | 20 (18-21) | 3 (21%) | 2 (14%) |
| **Executive function** |  |  |  |
| Trail Making Test B (sec) | 83 (63.25-138.75) | 9 (64%) | 4 (29%) |
| Semantic generative naming | 36 (25.75-38.75) | 1 (7%) | 0 (0) |
| COWAT phonemic | 26.5 (15.5-42) | 5 (36%) | 1 (7%) |

NMOSD, neuromyelitis optica spectrum disorders; SD, standard deviation; PASAT, Paced Auditory Serial Addition Test; K-BNT, Korean version of the Boston Naming Test; K-CVLT, Korean-California Verbal Learning Test; RCFT, Rey-Osterrieth Complex Figure Test; COWAT, Controlled Oral Word Association Test

Data area expressed as median (interquartile range)
